# Supplementary material for: Are carbon emissions trading and green financial instruments synergistic? -Comprehensive quantitative research based on content analysis
Source: PLoS One. 2024 Mar 7;19(3):e0298601. doi: 10.1371/journal.pone.0298601 (PMC10919601; doi:10.1371/journal.pone.0298601)
Supplement: S1 Data — (ZIP) [file pone.0298601.s001.zip › Data/地级市Z_F工作报告.zip!/╣π╢1⁄2/║╙╘┤╩╨/└┤╫╘╣1⁄2╓┌║┼í╛┬φ┐╦╩2╛▌═°í┐_2013.docx]

各位代表：

现在，我代表市人民政府向大会作政府工作报告，请予审议，并请各位政协委员和其他列席人员提出意见。

过去一年工作回顾

2012年，是我市经受住各种困难和风险考验，经济社会发展取得明显成绩的一年。新一届市政府在省委、省政府和市委的正确领导下，在市人大及其常委会和市政协的监督支持下，牢牢把握主题主线，以为人民幸福而发展为工作方向，突出“发展提速年”、“管理强化年”和“工作落实年”，团结带领全市人民沉着应对、攻坚克难，坚持“保增长、调结构、强基础、促转型、惠民生”，经济社会实现平稳较快发展，较好完成市六届人大一次会议确定的目标任务。

一、建平台、抓招商、上项目，生态经济凸显新亮点

把建平台、抓招商、上项目作为推动加快发展、转型升级的重要突破口，发展质量水平稳步提高。经济平稳较快增长。主要经济指标下半年实现企稳回升，增幅逐月加快。全市生产总值615.3亿元，比上年增长11.6%，增速排全省第5位；全社会工业总产值和增加值分别为1077亿元和260.4亿元，分别增长17.2%和18.1%，规模以上工业增加值增速排全省第8位；地方公共财政预算收入37.64亿元，增长20%，增速排全省第7位；固定资产投资278.6亿元，增长18%；社会消费品零售总额209.4亿元，增长13.3%；实际利用外资1.97亿美元，增长10.3%，增速排全省第5位；外贸出口19.5亿美元，增长1.9%；民营经济占全市生产总值比重达60.4%；节能降耗和污染减排任务完成。产业转型升级加快。实施国家、省、市科技计划项目513项，科技创新能力稳步提升；新一代移动通信终端制造产业基地成为省战略性新兴产业基地，手机整机产量突破6000万台，手机产业产值220亿元；源城区电子产业集群被认定为省产业集群升级示范区；一批矿产精深加工企业建成投产，矿业经济逐步从粗放开采向精深加工转变；13个项目入围广东现代产业500强项目。灯塔盆地成为国家现代农业示范区；建成省级以上现代农业示范区54个，新增省、市级农业龙头企业15家、绿色品牌15个，农民专业合作社总量居全省首位；农业总产值126.6亿元，增长6%。大力发展生态旅游，东江？巴登城、东江源休闲观光中心、康泉养生休闲度假区和希尔顿酒店等旅游重点项目顺利推进，接待游客总人数1650.6万人次、总收入132.4亿元，分别增长18.7%、116.7%。房地产、商贸流通等现代服务业发展良好。园区发展卓有成效。“一区六园”开发建设投入13.3亿元，配套设施不断完善，市高新区完成三、四期征地6.22平方公里，园区承载能力进一步提升。中山（河源）转移园在全省考评中名列第一、盐田（东源）转移园评为优秀；中山（河源）、罗湖（源城）转移园分别获评省五星级、三星级优秀园区。“一区六园”新签约项目74个，合同投资总额116亿元，完成工业总产值665.4亿元、增加值123.4亿元，分别增长24.9%、29.5%。成功举办“金秋河源？广东绿谷”等系列经贸活动，大力实施“乡贤反哺”工程，280亿元投资项目签约落户。重点项目稳步推进。省、市重点项目完成投资95.7亿元。汉能光伏、旗滨玻璃、中光电通讯等一批项目顺利投产；长江家俱、一通汽贸、连平钨矿深加工等一批项目落户。金融业持续快速发展。金融机构贷款余额473亿元，存贷比达75.37%。通过搭建政银企合作平台促成贷款61.5亿元，有力支撑实体经济发展。汇金典当公司成功在香港联交所上市，实现本地企业境外上市“零”的突破。引进首家外资银行汇丰银行在我市设立分支机构。完成43个乡村金融服务站和信用村试点建设，行政村银行卡助农取款服务点覆盖率100%。保险业加快发展,保费收入增长11.82%。

二、修路、治水、种树，宜居城乡建设取得新突破

坚持把修路、治水、种树作为优化发展环境的重中之重，大力建设宜居城乡。交通能源建设取得新进展。东环、大广、河紫3条高速公路顺利开工；实施市区兴源路、凯丰路、长堤中路改造升级，贯通市区永和路、纬十一路、纬十二路等“断头路”；环市路建设顺利推进；完成省、县、乡道路改造158公里，完成新农村公路路面硬化建设525公里。电网建设和农网改造年度任务完成，建成110千伏输变电工程3个、送电线路工程3个，新增35千伏及以上变电容量14.3万千伏安、送电线路51.6公里。国华金太阳、东源蝉子顶风电、和平大唐风能等新能源项目顺利推进。农田水利建设成效明显。投入3.8亿元，完成5条中小河流治理、25座重点小（2）型水库除险加固、5个中央小型农田水利重点县与高效节水灌溉、6个省级小型农田水利示范镇和连平县瓮潭灌区改造等工程建设任务。东源、紫金两县成功竞得省级水利建设示范县扶持资金共8.8亿元。生态环保建设稳步推进。积极创建林业生态市，完成森林碳汇重点生态工程人工造林种植38.95万亩，森林覆盖率达72.9%。新建污水处理厂2座，新丰江水库库区6个镇污水处理设施动工建设，东江教育城污水过江管网及泵站工程加快推进，高埔片区截污管网建成，万绿湖生态环境专项整治取得阶段性成效。城乡宜居水平不断提升。强化经营城市理念，优化城市功能政策，成功筹集城建资金18.5亿元。推进北连战略，市区连接东源县城征地拆迁基本完成。启动跨江战略。完成风光、钓鱼台、庄田片区等一批控制性详规和专项规划。顺利通过国家土地卫片检查，用地保障水平提升。改造升级一批农贸市场。市区公交线路增加。城乡环境卫生整治行动、宜居城乡“六个一”工程和“大清洁，乡村美”农村清洁工程扎实推进。新增18个省级古村落、47个省级卫生村，和平县及林寨古村落分别获评省旅游强县、广东十大最美古村落。

三、拆违建、打偷采、强管理，善治河源打开新局面

对河源发展高度负责，依法打击“三违四抢”和非法采矿行为，大力破解发展难题和历史难题。专项整治成效显著。开展了严厉打击“三违四抢”专项行动，依法拆除违法建（构）筑物5179宗、108.3万平方米，有效遏制“三违四抢”行为；开展了打偷采专项行动，查处非法矿点137个，复绿1364.4亩，没收稀土矿产品2404.3吨；开展源城区农村宅基地调查摸底及建筑物甄别界定工作，完善了长效监管机制。“三打两建”深入推进。“三打”工作卓有成效，摸排线索11287起，查处涉案人员14850人，打掉“保护伞”293个，涉案财物价值6.64亿元。社会信用体系和市场监管体系建设积极推进。社会管理不断加强。深入开展公安机关“八大会战”，深化社会管理“六项创新”，积极实施“六五”普法规划和开展人民调解，社会治安综合治理和信访维稳工作进一步得到加强。全面落实安全生产“一岗双责”制度，安全生产形势总体稳定。产品质量和食品药品安全监管、救灾减灾、应急管理等工作得到加强。积极做好“2？16”地震抗震工作。创建一批省级餐饮服务食品安全示范街（镇）。

四、强教育、兴文化、塑文明，文化河源呈现新气象

坚持教育优先，大力提升文化软实力，积极创建全国文明城市。教育创强快速推进。21个镇和源城区实现创强目标。新建、改建镇、村幼儿园 127所，建设义务教育规范化学校 67所，新建3所民办学校，高中阶段教育毛入学率达87.8%，小学和初中学龄人口入学率达99.97%和99.66%。新建4所特殊教育学校，特殊学校标准化建设加快。文化事业不断进步。完成市第三次全国文物普查，新增省级文物保护单位4处。建成3家县区数字影院，完成110家行政村文化室公共电子阅览室建设，新建442家农家书屋，广播电视村村通工程全面完成。“三馆一站”实现免费开放。成功举办中国第二届客家文化节和市第九届客家文化旅游节。完成国有文艺院团体制改革。紫金县花朝戏剧团、连平县文化馆成为省首批非物质文化遗产传承基地。知识产权、档案和地方志工作得到加强。完成市直机关使用正版软件工作任务。成功举办市第三届运动会，我市激流回旋选手打破我国参加奥运会该项目纪录。文明创建全面铺开。启动“四馆两中心”建设，创建一批“文明行业”、“文明单位”、“文明镇”、“文明村”、“文明社区”。源城区埔前镇泥金村社区和紫金县紫城镇通惠社区获评“全国防灾减灾示范社区”。开展了“多彩河源，欢乐广场”等群艺活动。成功再创“全国双拥模范城”。

五、保民生、促脱贫、办实事，人民生活得到新改善

坚持把保障和改善民生作为政府工作的根本目的，切实解决好关系人民群众切身利益的突出问题，不断提升人民群众幸福感。民生保障水平提高。基本民生、底线民生和热点民生得到切实保障。新增城镇就业岗位4.8万个，农村劳动力技能培训3.5万人，新增劳动力培训转移就业5.4万人，城镇登记失业率控制在2.3%以内。城镇居民可支配收入16520元、增长12.1%，农民人均纯收入7772元、增长15.4%。城乡居民参加养老保险114.6万人、新农合283万人、基本医疗保险63万人，五大险种参保人数增长4%以上、基金累计结余超30亿元。建立80周岁以上老龄津贴制度。落实65周岁以上老人乘坐公交车优惠政策。开展“善治河源”助学助医活动。农村五保供养水平大幅提高。低保救济面占全市人口7%。建成农副产品平价商店82家。医改稳步推进，公办基层医疗机构全部实施国家基本药物和零差率销售制度。加大基层医疗机构投入，积极推进基层医疗机构综合改革。市中医院实现搬迁开业，县级中医院6个专科通过国家农村中医特色专科建设评审。实施优生健康惠民工程，全面完成人口计划，较好稳定低生育水平。扶贫“双到”目标全面实现。投入扶贫“双到”资金7.14亿元，318个贫困村集体经济年均收入8.89万元，4.21万户贫困户人均年纯收入8300元，完成低收入住房困难户住房改造2.17万户。建成省（东源）水库移民“双转移”示范基地一期工程。移民生产生活水平不断提高。“十件实事”顺利完成。投入民生资金102.5亿元，市博爱学校、市卫生学校一期工程、河源理工学校实训中心三期、河职院实训中心三期、河源中学改建工程竣工使用，市体校迁建工作启动；市区道路升级改造顺利推进；迎客大桥和滨江大道二期开工建设；客家文化公园首期工程建成，市博物馆、市图书馆新馆建设前期工作进展顺利；人力资源社会保障公共服务平台全面建成；农村饮水安全工程建设总任务基本完成；保障性安居工程顺利推进；生态景观林带示范段建设任务超额完成；市人民医院新院二期和市妇幼保健院新院二期建设工程顺利启动。

六、转作风、优服务、抓落实，行政效能再上新台阶

坚持优化政府服务，转变作风，狠抓落实，不断增强政府执行力，牢牢把握工作主动权。坚持依法行政，自觉接受市人大及其常委会的法律监督和市政协的民主监督，人大建议、政协提案办复满意和基本满意率100%。开展作风建设年活动，积极治理个别部门领导干部“庸、懒、散、浮、奢”等不良现象。全面开展“三赛”和“工作落实年”活动，出台了工业园区和重点项目建设等考核办法，健全了工作落实机制。积极开展行政审批制度改革。加大扶持企业发展力度，帮助企业解决发展难题，减免中小微企业税费1.6亿元；实施领导挂钩服务企业制度，投资者对政府评价进一步提高。全面落实党风廉政建设责任制，推进政务公开和网络问政，确保行政权力规范和阳光运行。推动公共资源交易平台建设，对土地交易、产权交易、建设工程招投标和政府采购领域实施实时监督管理。

此外，法律援助、“双拥”创建、优抚安置、人事编制、民族宗教、行政服务、驻外机构、外事侨务、妇女儿童、人民武装、审计、科普、对台、人防、气象、打私、残疾人等各项工作取得新进展。

各位代表！这些成绩来之不易，弥足珍贵。这是省委、省政府和市委正确领导的结果，是全市人民共同奋斗的结果，也离不开广州、深圳、中山等兄弟市和海内外各界人士的关心支持。在此，我代表市人民政府，向全市广大干部群众、驻河源部队、消防官兵和各民主党派、人民团体、社会各界人士致以崇高的敬意！向关心支持我市改革发展的兄弟市以及港澳同胞、台湾同胞、海外侨胞表示衷心的感谢！

我们也清醒地认识到，我市经济社会发展还存在不少困难和问题。主要表现在：受大环境等因素影响，部分目标未能如期实现；经济总量仍然较小，工业经济和县域经济发展较慢，工业园区接纳能力还不够强，支撑经济发展作用有所减弱；好项目、大项目不多，项目签约多、落户少；资源制约日益明显，生态保护和节能减排任务更加艰巨；城镇化水平滞后，城乡发展不够协调；财政收支矛盾突出，改善民生任务相当繁重；部分部门及干部服务意识淡薄，行政效能和服务水平有待提升。对此，我们要高度重视，采取措施，认真予以解决。
